# Impact of Different Gums on Textural and Microbial Properties of Goat Milk Yogurts during Refrigerated Storage

**DOI:** 10.3390/foods8050169

**Published:** 2019-05-18

**Authors:** Young W. Park, Jolethia Oglesby, Saeed A. Hayek, Sulaiman O. Aljaloud, Rabin Gyawali, Salam A. Ibrahim

**Affiliations:** 1 Georgia Small Ruminant Research and Extension Center, Fort Valley State University, The University System of Georgia, Fort Valley, GA 31030, USA; parky@fvsu.edu (Y.W.P.); jolethiao@yahoo.com (J.O.); 2 Food Microbiology and Biotechnology Laboratory, Food and Nutritional Sciences Program, North Carolina Agricultural and Technical State University, Greensboro, NC 27411, USA; safesaeed@yahoo.com (S.A.H.); saljaloud@ksu.edu.sa (S.O.A.); ibrah001@ncat.edu (S.A.I.)

**Keywords:** caprine milk, gums, xanthan, locust bean, yogurt properties, refrigeration

## Abstract

In this study, the impact of seven different gums on textural and microbiological properties of goat milk yogurt during refrigerated storage was investigated. The results showed that yogurt containing xanthan and locust bean gums had enhanced firmness, consistency, cohesiveness, and viscosity during four weeks of storage compared to the control and yogurt fortified with other gums (*p* < 0.05). The addition of gums also helped to maintain the microbial viability of the yogurt culture and the probiotic *Bifidobacterium* spp. This study thus demonstrated that these gums could be used in the production of goat milk yogurt with enhanced textural properties.

## 1. Introduction

Yogurt is a popular fermented dairy food in many parts of the world, especially in Europe, North America and the Middle East. The high consumption and popularity of yogurt, particularly among women, children and teenagers, are primarily due to its nutritional, health and therapeutic attributes [1,2]. Yogurt is produced by the fermentation of milk with bacterial cultures that consist of a mixture of *Streptococcus* subsp. *thermophilus* and *Lactobacillus delbrueckii* subsp. *bulgaricus.* Curd texture, or firmness, is an important property of yogurt and determines the quality and acceptability of the product. Adequate firmness without syneresis is also essential for improving the quality of yogurt [3]. The milk curd of yogurt is formed with the acid produced by lactic acid bacteria. This result is a consequence of removing calcium and neutralizing the negative charges of casein micelles which, in turn, causes destabilization of casein that aggregates and forms a curd [4]. 

Goat milk has been recommended as an alternative milk for people allergic to cow’s milk. In addition, goat milk has a higher amount of small fat globules that are very important in human nutrition, especially for children and the elderly [5]. As a result, there is an increasing demand for goat milk products, such as yogurt. However, goats’ milk produces a softer curd during the fermentation process and is characterized by less firm gel and lower viscosity than its cow milk counterpart [6]. Therefore, it is difficult to make goat milk yogurt with a consistency comparable to that of cow milk yogurt, which is primarily due to the difference in casein content and its composition [7]. Several methods have been applied to improve consistency and/or texture of yogurt. The most commonly used methods include an increase of total solids in the milk and the addition of gums as stabilizers [8,9,10,11]. Gums are polysaccharides of natural origin that increase viscosity in solution, even with small concentrations. Pectin’s, anionic charged polysaccharides derived from plant cells of fruit, are often used as gelling agents and stabilizers in low-pH food products, such as yogurt [12]. Xanthan gum has been used to improve the texture, increase firmness and prevent syneresis in yogurt [13]. Similarly, Carrageenan, synergistic with some other gums, can increase the gel strength and water-binding capabilities, as well as modifying the gel texture of yogurt [14]. Other gums, such as locust bean and guar, also increased the viscosity and gel strength and can be applied in yogurt to improve the textural characteristics [10]. 

Probiotics are live microbial food supplements that benefit the health of consumers [15] or live microorganisms that, when administered in adequate amounts, confer a beneficial effect on the host [16]. The best-known probiotics are *Lactobacillus acidophilus*, *Lactobacillus casei*, *Lactobacillus plantarum*, and bifidobacterial species which are used in yogurt and other dairy products. These beneficial bacteria retain viability during storage and survive passage through the stomach and small bowel. Thus, probiotics have been consumed as functional foods because they provide health benefits beyond the traditional nutrition function [17]. A number of lactic acid bacteria (LAB), propionibacteria and bifidobacteria can synthesize exopolysaccharides (EPS), which are excreted from the cell and which may or may not be loosely attached to the cell wall [18]. EPS can contribute to the improved stability, rheology and texture of fermented dairy products and may also offer protection to cells against phage attack, desiccation and osmotic stress [19]. The levels of EPS that are formed are dependent on growth phase, temperature, pH and carbohydrate source and are not a direct function of growth [20]. However, the effects of natural gum supplementations on textural properties and microbial viability of goat milk yogurt have been studied very little. Therefore, the aims of the present investigation were to: (1) Evaluate the textural characteristics of goat milk yogurt supplemented with seven different gums; and (2) determine the effects of these gums on the microbial viability in goat milk yogurt during four weeks of refrigerated storage.

## 2. Materials and Methods

### 2.1. Experimental Design

The experiment was conducted as randomized block design. Three batches of plain goat milk yogurt was manufactured. Yogurt starter culture (*Streptococcus* subsp. *thermophilus* and *Lactobacillus delbrueckii* subsp. *bulgaricus*) and probiotic *Bifidobacterium animalis* Bb12 strain were obtained from Chr. Hansen Inc. (Milwaukee, WI, USA). Stock cultures of these bacteria were maintained at −80 °C in Food Microbiology and Biotechnology Laboratory at North Carolina A&T State University (Greensboro, NC, USA). Seven different gums: (i) Xanthan; (ii) modified food starch with agar pectin; (iii) carrageenan; (iv) locust bean; (v) carrageenan, maltodextrin and dextrose; (vi) guar; and (vii) modified food starch with gums 3, 4 and 5 (provided by TIC gums, Belcamp, MD, USA) were added to the pasteurized goat milk before manufacture of the yogurt. Experimental yogurt samples were manufactured in triplicate for control and yogurt with each of the seven different gums. From each replicate of each batch, samples were collected and analyzed at 0, 2 and 4 weeks during a refrigerated (4 °C) storage.

### 2.2. Preparation of Goat Milk

The caprine milk used in the study was bulk tank milk collected from the mid lactation milking goat herd consisting of Saanen, Alpine and Toggenburg breed at the Georgia Small Ruminant Research and Extension Center, Fort Valley State University, Fort Valley, Georgia, USA. All milking goats were pasture fed at the university farm, and each animal was fed a supplement of one pound (0.454 kg) of concentrate feed per day. 

### 2.3. Manufacture of Goat Milk Yogurts

The experimental caprine milk yogurt was manufactured using pasteurized goat milk heated to 85 °C for 30 min and cooled to 45 °C. Five g of each individual gum was added to 1 L of milk and mixed after which 25 mL of the activated bacterial culture containing yogurt culture (*L. bulgaricus* and S. *thermophilus*) and the probiotic *Bifidobacterium* Bb12 was inoculated to the milk. The milk samples were filled to 10 oz plastic cups in triplicates. The containers were incubated at 45 °C until the pH reached 4.6 (~4 h). The yogurt was then stored at 4 °C, and texture and microbial viability were performed for each sample.

### 2.4. Texture Analysis of Yogurts

Textural characteristics of all yogurt samples were determined using a texture analyzer (Model TA.XT2i, Texture Technology Corp., Scarsdale, NY, USA), equipped with 5 kg head. A cylindrical probe was used for detecting shear force (g) which was made of acrylic material and 2.5 cm in diameter and 3.5 cm in height. The speed of the probe was set at 2 mm/s as the recommended procedure of Guinard et al. [21]. The viscosity of the yogurt was measured by firmness (g force) and consistency. The adhesiveness or stickiness of the samples was measured by cohesiveness (g force) and index of viscosity. 

### 2.5. Microbial Viability Assay

Throughout the storage period, ten ml of each yogurt sample was diluted with 90 mL of sterile peptone water (0.1%), mixed well, and serial dilutions were made. A 0.1 mL sample of the appropriate dilutions was surface plated onto an agar medium of deMan Rogosa and Sharpe (MRS), M-17, and Bifidobacterium Iodoacetate (BIM-25) for enumerating populations of *Lactobacillus bulgaricus*, *S. thermophilus*, and *Bifidobacterium* spp., respectively. The inoculated plates were incubated at 40 °C for 48–72 h, and then colonies were counted [22]. 

### 2.6. Statistical Analysis

Statistical analyses were performed using a general linear model procedure of SAS program (SAS Inst., Cary, NC, USA). The Analysis of Variance (ANOVA) test followed by Duncan’s multiple-range test was used to determine significant differences in the means. A *p*-value of < 0.05 was considered statistically significant.

## 3. Results and Discussion

### 3.1. Textural Characteristics

The results of the textural properties of control and seven different gums supplemented goat milk yogurt are summarized in Table 1. As shown in the table values, the xanthan and locust bean gums showed distinctively and significantly (*p* < 0.05) higher firmness in the yogurt than control and other types of gum-supplemented groups. This would indicate that xanthan and locust bean gums possessed higher textural binding abilities than the other gums with regard to forming firmer yogurt texture. The differences in yogurt firmness between the control and other gum groups were not significant. In a study of cow milk yogurt, El-Sayed et al. [13] observed that fortification of xanthan gum in the yogurt improved the texture, increased the firmness and prevented syneresis. Ertan et al. [14] reported that carrageenan had a synergistic effect with some other gums in increasing the gel strength, water-binding capacities and modification of the gel texture in cow milk yogurt. These results show that supplementation with gums can improve the firmness, consistency and cohesiveness of fermented foods, especially yogurt. 

Refrigerated storage treatments resulted in a tendency toward increases in firmness in proportion to the storage time. Regardless of the type of gum supplementation, and including the control group, the firmness generally increased, as shown in Table 1. The elevations in firmness for xanthan and locust bean gum added yogurt was significantly greater than the other yogurt groups. These outcomes suggest that cold storage of the goat yogurt may also improve the firmness of the product as the increases in firmness of the control and other gum-fortified groups were minimal and insignificant. 

With regard to the experimental goat yogurt, the xanthan and locust bean supplemented yogurt also showed significantly (*p* < 0.05) greater consistency than that of the control and other gum-added yogurt. These results clearly suggest that xanthan and locust bean supplementation in the goat milk yogurt produced the highest textural integrity among all tested gums and could thus be used to enhance the texture of caprine yogurt. Zhang et al. [23] also found that the added xanthan gum or carrageenan increased the firmness, consistency and cohesiveness of the low-fat fermented skim milk ST0 or ST1. Griffin et al. [24] reported that commercial goat milk yogurt supplemented with tapioca had significantly (*p* < 0.05) greater firmness and consistency than commercial cow milk yogurt and plain goat milk yogurt without any supplementation. Tapioca is basically a root starch derived from the cassava, or yuca plant. It is often used to thicken soups and sweeten the flavor of baked goods and was used in the commercial goat milk yogurt to thicken the product. 

An interesting trend was observed with regard to consistency values of the xanthan and locust bean fortified yogurt during the storage periods. Although both gum-added yogurt showed significantly (*p* < 0.05) higher consistency than the other gum-added groups, xanthan yogurt had a decrease in consistency values as the storage period advanced, while the locust bean yogurt showed the opposite trend during the four weeks of refrigerated storage. However, of the experimental goat yogurt, xanthan gum had higher values than locust bean gum in overall consistency.

With regard to viscosity, cohesiveness and adhesiveness of the tested caprine yogurt, xanthan and locust bean supplemented goat yogurt also showed significantly (*p* < 0.05) higher values (cohesiveness and index of viscosity) than the control and the other gum supplemented goat yogurt. All tested textural properties in our study revealed that xanthan and locust bean are the superior choice of gums for improving the texture of the caprine milk yogurt. 

### 3.2. Viability of Bacterial Populations during Storage

Changes in the bacterial populations in goat yogurt fortified with seven different gums during the four weeks of storage are shown in Table 2. In general, *L. bulgaricus* counts were highest at the beginning of the storage period and decreased throughout the storage periods in all gum-fortified yogurt and the control group. For non-fortified control samples, the bacterial populations decreased from initial counts of 8.47 log CFU/mL to 8.09 log CFU/mL after four weeks in refrigerated storage. With the addition of gums, the bacterial population reached to 8.16–8.65 log CFU/mL from the initial count of 8.26–8.89 log CFU/mL. In the presence of gums, the reduction level of the bacterial population was similar to that of the control sample after four weeks of storage. Although the differences were not statistically significant (*p* > 0.05), there was a general trend toward a decrease in the bacterial populations in proportion to the storage time period in all treatment groups. 

Similarly, in the control sample, there was an almost 1 log reduction in *S. thermophilus* counts when compared to the initial population of 7.04 log (day 0) with 6.10 log (four weeks) However, in the presence of gums, we observed less than 1 log reduction in bacterial counts in the samples fortified with gums at the end of the storage period. Bacterial populations were in the range of 7.00–7.99 log in the treated samples, indicating that gums could maintain a slightly higher viability of *S. thermophilus*. Results showed that all the tested gums maintained the viability, but did not provide additional protection for the bacterial population. 

In the case of probiotic *Bifidobacterium* spp., bacterial counts decreased from initial populations of 7.7 log (day 0) to 5.02 log (four weeks) in the control sample. Yogurt fortified with gums showed good viability at the end of the storage period compared to the control sample. At the end of the storage period, probiotic viability was slightly better, but not significantly different (*p* > 0.05) in yogurt fortified with gums # 4 (locust), 5 (carrageenan), and 6 (Guar) compared to the control and others. Karlton-Senaye et al. [25] reported a higher viable number of probiotic *Lactobacillus rhamnosus* GGB103 in the product containing gum # 5 (carrageenan–maltodextrin). Similarly, Ghasempour et al. [26] reported that the viability of *L. acidophilus* and *B. bifidum* of yogurt during 29 days of refrigerated storage was greater in yogurt fortified with gums, such as carrageenan and guar. These results thus indicate that these gums could maintain the viability of probiotic bacteria. Since enhanced viability is necessary for probiotic functionality, food products should contain adequate levels of probiotics throughout the storage period. Therefore, these gums could serve as good medium for dietary incorporation of probiotics.

## 4. Conclusions

Among the seven gums tested, locust bean and xanthan would be the most desirable gums for achieving higher textural quality in the manufacture of caprine milk yogurt. Even though these gums did not show any significant effect on bacterial viability, locust bean and xanthan gums could be promising stabilizers to enhance the rheological properties of caprine milk yogurt. 

## Figures and Tables

**Table 1:** Summary of the textural property of yogurt during refrigerated storage.

Sample	Storage Period	Firmness		Consistency		Cohesiveness		Index of Viscosity	
		X	SD	X	SD	X	SD	X	SD
Control	0 Day	19.05	1.54	67.80	4.65	5.33	0.13	3.81	1.36
	2 Weeks	21.15	0.84	46.16	2.08	6.00	0.45	2.96	0.86
	4 Weeks	22.99	1.08	49.33	1.54	6.22	0.27	2.75	0.45
Gum 1	0 Day	41.84 *	9.93	172.04 *	94.97	6.42 *	1.38	5.42 *	2.46
	2 Weeks	73.43 *	14.37	147.74 *	47.40	12.40 *	3.72	10.78 *	3.77
	4 Weeks	63.69 *	10.29	114.16 *	19.05	11.54 *	2.18	9.71 *	1.88
Gum 2	0 Day	20.58	1.71	45.13	3.61	5.40	0.15	1.49	0.07
	2 Weeks	23.19	2.43	49.24	4.44	6.73	0.58	3.50	0.94
	4 Weeks	22.23	1.11	46.16	4.39	6.48	0.56	2.93	0.51
Gum 3	0 Day	19.67	1.66	43.33	3.29	5.00	0.19	1.59	0.16
	2 Weeks	20.59	0.94	44.16	1.38	5.83	0.67	2.49	0.41
	4 Weeks	20.91	0.86	45.00	0.54	5.66	0.40	2.11	0.26
Gum 4	0 Day	50.21 *	7.10	100.51 *	20.11	8.10 *	1.24	6.10 *	1.16
	2 Weeks	55.40 *	3.20	108.88 *	8.54	11.69 *	3.04	10.1 *	2.30
	4 Weeks	64.68 *	7.13	132.39 *	12.72	15.77 *	1.15	15.21 *	0.89
Gum 5	0 Day	18.90	0.56	41.54	1.29	5.13	0.39	1.40	0.13
	2 Weeks	19.60	0.75	43.32	0.80	5.16	0.78	1.74	0.17
	4 Weeks	19.71	0.80	42.58	1.09	5.25	0.35	2.02	0.34
Gum 6	0 Day	20.00	1.24	42.89	2.43	4.81	0.21	1.58	0.22
	2 Weeks	20.20	1.56	43.10	3.22	5.23	0.47	2.08	0.36
	4 Weeks	20.84	0.50	44.19	1.01	5.28	0.20	1.96	0.35
Gum 7	0 Day	19.33	0.26	42.64	1.38	5.19	0.15	1.84	0.11
	2 Weeks	20.67	0.74	44.90	1.42	6.06	0.30	2.58	0.13
	4 Weeks	21.09	0.64	44.03	2.73	5.82	0.23	2.02	0.06

X = Mean, SD = Standard deviation. Values are the mean ± SD (*n* = 3). * Significant differences were detected at *p* < 0.05 compared to the control and other gums. Gum 1 added yogurt had higher consistency value among all treatment groups, while gum 4 added yogurt has the highest values of all other textural values among all treatment groups (*p* < 0.05). Gums 1, 2, 3, 4, 5, 6 and 7 stand for: Xanthan, modified food starch with agar pectin, carrageenan, locust bean, carrageenan, maltodextrin and dextrose, guar, and modified food starch with gums 3, 4 and 5, respectively.

**Table 2:** The changes in bacterial population (Log CFU/mL) of yogurt during refrigerated storage.

Yogurt	Storage Period	*L. bulgaricus*	*S. thermophilus*	*Bifidobacterium* spp.
Control	0 Day	8.47 ± 0.27	7.04 ± 0.13	7.70 ± 0.08
	2 Weeks	8.13 ± 0.13	6.55 ± 0.09	6.21 ± 0.52
	4 Weeks	8.09 ± 0.47	6.10 ± 0.15	5.02 ± 0.45
Gum 1	0 Day	8.26 ± 0.11	7.00± 0.17	7.81 ± 0.22
	2 Weeks	8.19 ± 0.30	7.26 ± 0.32	7.14 ± 0.17
	4 Weeks	8.23 ± 0.66	7.49 ± 0.52	5.68 ± 0.34
Gum 2	0 Day	8.84 ± 0.12	7.20 ± 0.31	7.79 ± 0.24
	2 Weeks	7.83 ± 0.20	7.02 ± 0.19	7.01 ± 0.33
	4 Weeks	8.16 ± 0.16	7.00 ± 0.21	5.67 ± 0.44
Gum 3	0 Day	8.61 ± 0.09	7.10 ± 0.18	7.74 ± 0.30
	2 Weeks	8.22 ± 0.17	7.28 ± 0.14	6.85 ± 0.29
	4 Weeks	8.20 ± 0.10	7.10 ± 0.52	5.56 ± 0.18
Gum 4	0 Day	8.54 ± 0.20	7.14 ± 0.39	7.60 ± 0.41
	2 Weeks	8.40 ± 0.14	7.70 ± 0.40	7.32 ± 0.18
	4 Weeks	8.39 ± 0.33	7.41 ± 0.66	5.85 ± 0.27
Gum 5	0 Day	8.61 ± 0.49	7.78 ± 0.72	7.10 ± 0.10
	2 Weeks	8.48 ± 0.35	8.08 ± 0.48	6.91 ± 0.33
	4 Weeks	8.32 ± 0.47	7.39 ± 0.11	5.45 ± 0.45
Gum 6	0 Day	8.75 ± 0.19	7.90 ± 0.17	7.60 ± 0.55
	2 Weeks	8.50 ± 0.23	8.03 ± 0.09	6.79 ± 0.38
	4 Weeks	8.36 ± 0.41	7.34 ± 0.10	5.71 ± 0.19
Gum 7	0 Day	8.89 ± 0.11	7.91 ± 0.26	7.90 ± 0.17
	2 Weeks	8.70 ± 0.21	8.04 ± 0.31	7.45 ± 0.27
	4 Weeks	8.65 ± 0.15	7.99 ± 0.29	5.98 ± 0.31

Values are the mean ± SD (*n* = 3). There were no significant differences (*p* > 0.05) in bacterial population compared to the control and other gums. Gums 1, 2, 3, 4, 5, 6 and 7 stand for: Xanthan, modified food starch with agar pectin, carrageenan, locust bean, carrageenan, maltodextrin and dextrose, guar, and modified food starch with gums 3, 4 and 5, respectively.
